# Anatomical Study and Classification of Foramina of the Squamous Part of the Temporal Bone

**DOI:** 10.1002/ca.70024

**Published:** 2025-09-05

**Authors:** Tawanrat Paensukyen, Pattita Kanjanapaisan, Napawan Taradolpisut, Nutmethee Kruepunga, Benrita Jitaree, Pakpoom Thintharua, Arada Chaiyamoon, Athikhun Suwannakhan

**Affiliations:** ^1^ Biomedical Science Program, Faculty of Science Mahidol University Bangkok Thailand; ^2^ Department of Anatomy, Faculty of Science Mahidol University Bangkok Thailand; ^3^ Department of Anatomy, Faculty of Medicine Kasetsart University Bangkok Thailand; ^4^ Chakri Naruebodindra Medical Institute, Faculty of Medicine, Ramathibodi Hospital Mahidol University Samut Prakan Thailand; ^5^ Department of Anatomy, Faculty of Medicine Khon Kaen University Khon Kaen Thailand; ^6^ Department of Biomedical Sciences, College of Medicine and Health University of Birmingham Birmingham UK

## Abstract

The postglenoid foramen (PGF) is a rarely reported anatomical variant of the temporal bone, with limited data on its morphology, prevalence, and clinical relevance, particularly in non‐European populations. This study aimed to investigate the anatomy and frequency of foramina located on the squamous part of the temporal bone, including the PGF, and to propose a classification system based on their anatomical positions. A total of 117 human skulls (234 sides), including both dried and Thiel‐embalmed specimens, were examined through gross observation, cadaveric dissection, and computed tomography (CT) in selected cases. Foramina were localized relative to the postglenoid process (PGP) and zygomatic arch, and classified accordingly. Foramina were identified in 48 skulls (41.0%) and 67 sides (28.6%), with no significant difference between left and right sides (*p* = 1.00). Based on anatomical location, three main types were identified: preglenoid (76.7%), postglenoid (6.8%), and supra‐arcuate (16.4%). Three foramina were found to communicate with the middle cranial fossa, confirmed by CT imaging. The proposed classification provides a practical framework for describing their positional variation. These findings are of clinical importance, particularly in surgical planning and radiologic interpretation, as such foramina may serve as conduits for venous communication or infection. Further studies are warranted to explore their histological structure and developmental origins.

## 1 Introduction

The temporal bone is a delicate and complex structure forming the lateral wall and base of the skull. It houses critical components, including the auditory and vestibular systems, and transmits several important neurovascular structures such as the facial nerve (CN VII), vestibulocochlear nerve (CN VIII), middle meningeal artery, and labyrinthine artery (Isaacson 2018; Drake et al. 2009). The external surface of the temporal bone is generally considered one of the most consistent anatomical regions of the human body. The only commonly documented variation is the postglenoid foramen (PGF), a rare opening located posterior to the glenoid fossa.

The PGF is a rare emissary foramen (Priya and Yasodai 2020) located anterior to the external acoustic meatus. Embryologically, it serves as a secondary drainage route for the petrosquamous sinus (PSS), which normally regresses before birth (Priya and Yasodai 2020). However, the PSS may persist into adulthood, functioning as an alternative drainage channel from the transverse sinus. The prevalence of PSS in adults is approximately 11.1% (Pękala et al. 2018). Clinically, the PGF is often overlooked during surgical procedures involving the temporal bone, increasing the risk of complications such as bleeding (Aderval Aragão et al. 2021). The persistence of the PSS has also been associated with auditory symptoms, including tinnitus (Alsherhri et al. 2011).

The PGF exhibits variability in its anatomical position. Most often, it is found anterior to the external acoustic meatus (Aderval Aragão et al. 2021). Butler (1957) described it as forming between the squamous portion of the temporal bone and the tympanic ring, while Wysocki (2002) identified it at two different locations: posterior to the glenoid fossa and superior to the zygomatic arch. Priya and Yasodai (2020) reported the PGF within the glenoid fossa. These findings suggest positional diversity of the PGF on the squamous part of the temporal bone, warranting a classification system that encompasses all observed variations. Furthermore, most prior studies have been conducted on European populations, with no data available from Southeast Asia, including Thailand.

This study aimed to investigate the anatomy and prevalence of foramina on the squamous part of the temporal bone, not only limited to the PGF, and to propose a comprehensive classification system based on their anatomical variations.

## 2 Materials and Methods

This study was approved and exempted by the Mahidol University Central Institutional Review Board (MU‐CIRB) (document number: COE No. MU‐CIRB 2025/061.1004). A total of 117 skulls (234 sides), including 97 dried skulls and 20 Thiel‐embalmed cadaveric skulls, were obtained from the Department of Anatomy, Faculty of Science, Mahidol University. Only skulls with intact temporal bones were included; those with fractured or incomplete temporal bones were excluded.

### 2.1 Observation of the Foramen, Morphometric Measurement, and Computed Tomography

The temporal regions (left and right) of all skulls were examined for the presence of foramina on the outer surface of the squamous part and the zygomatic process of the temporal bone. A 0.2 mm probe was inserted into each visible foramen to confirm patency; if the probe could not pass through, the foramen was excluded from further analysis. Discrepancies between two primary observers were resolved by a third observer.

Each foramen was localized using the postglenoid process (PGP) and zygomatic arch as anatomical landmarks. The position was classified as anterior, posterior, superior, inferior, or directly at the PGP. Other positions were recorded on a case‐by‐case basis. Distances from the PGP and the zygomatic arch were measured using a ruler. Locations anterior to the PGP were assigned negative (−) values, while those posterior were marked positive (+). Similarly, distances superior to the zygomatic arch were recorded as positive (+) and inferior as negative (−). Measurements were taken twice by two observers and remeasured three additional times to ensure accuracy. In cadaveric specimens, soft tissue around the temporal bone was dissected to expose the region of interest. The periosteum and connective tissue were removed to facilitate clear observation.

Computed tomography (CT) was performed on selected specimens using an uCT 350 scanner (United Imaging Healthcare Co. LTD) with a slice thickness of 0.55 mm.

### 2.2 Data Collection and Statistical Analysis

Prevalence was reported using descriptive statistics. Crude prevalence was defined as the number of skulls with at least one foramen divided by the total number of skulls. True prevalence was calculated as the number of sides with a foramen divided by the total number of sides. Descriptive statistics, including mean, standard deviation (SD), minimum, and maximum values, were calculated for the positional measurements. Left‐ and right‐sided prevalences were compared using the Chi‐square test, with a *p*‐value < 0.05 considered statistically significant.

## 3 Results

Among the 117 skulls (234 sides) examined, foramina were observed in 48 skulls (41.0%) (Table 1), including two cadaveric skulls. Of these, 29 skulls exhibited unilateral foramina, and 19 showed bilateral presence. A total of 67 foramina were found (28.6% of all sides), comprising 34 on the left and 33 on the right, with no significant side difference (*p* = 1.00). A single foramen was present on 67 sides, while six sides showed double foramina.

**TABLE 1:** Crude and true prevalence of the postglenoid foramen.

Prevalence	Left	Right	Prevalence (%)
Crude prevalence (skulls)	N/A	N/A	48/117 (41.0%)
Unilateral	N/A	N/A	29/117 (24.8%)
Bilateral	N/A	N/A	19/117 (16.2%)
True prevalence (sides)	34/234	33/234	67/234 (28.6%)

Abbreviation: N/A, not applicable.

**FIGURE 1:**
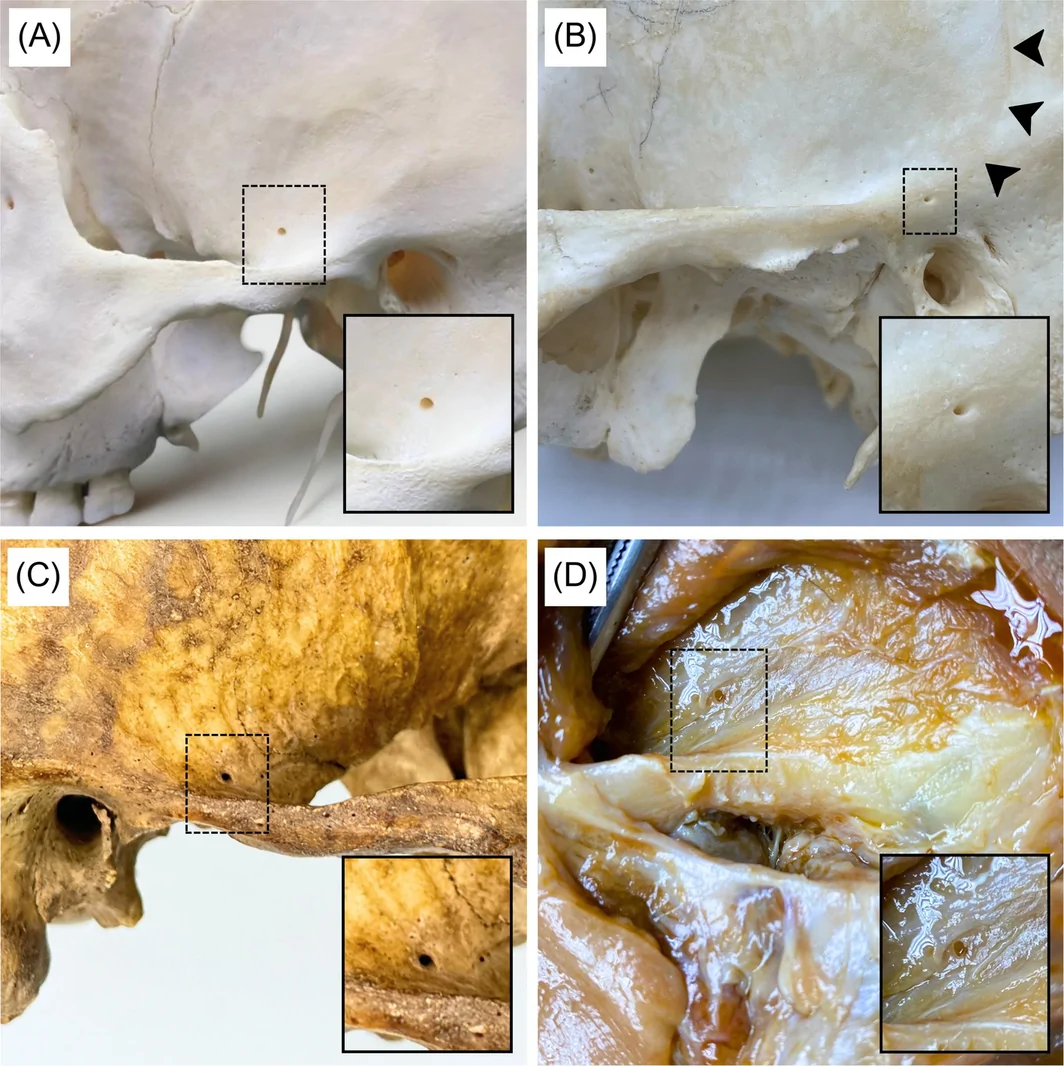
Classification of the foramina observed in the squamous part of the temporal bone including (A) preglenoid type, (B) postglenoid type, (C) supra‐arcuate type, and (D) dissection of Thiel‐embalmed cadaver showing the preglenoid type. The arrow heads indicate a body groove associated with the foramen.

Based on location, the foramina were categorized into three types: preglenoid, postglenoid, and supra‐arcuate (Table 2). Preglenoid type (anterior to the PGP and superior to the zygomatic arch) was the most common, present in 56 cases (76.7%). Postglenoid type (posterior to the PGP and superior to the zygomatic arch) was observed in 5 cases (6.8%). Supra‐arcuate type (lateral to the squamous part of the temporal bone) was found in 12 cases (16.4%). The preglenoid foramina were, on average, located 1.3 ± 0.3 cm anterior to the postglenoid process and 0.4 ± 0.2 cm superior to the zygomatic arch. The postglenoid type was found approximately 0.3 ± 0.1 cm posterior to the postglenoid process and 0.5 ± 0.1 cm superior to the suprameatal line. The supra‐arcuate type was situated about 1.5 ± 0.4 cm anterior to the postglenoid process, following the course of the suprameatal crest. In some cases where a foramen was observed, an associated bony groove was also evident (Figure 1B).

**TABLE 2:** Classification of postglenoid foramina based on anatomical position, showing prevalence and average location relative to the postglenoid process and zygomatic arch.

Types	Prevalence (%)	Average location
Preglenoid	56/73 (76.7%)	1.3 ± 0.3 cm anterior to the postglenoid process and 0.4 ± 0.2 cm superior to the zygomatic arch
Postglenoid	5/73 (6.8%)	0.3 ± 0.1 cm posterior to the postglenoid process and 0.5 ± 0.1 cm superior to the suprameatal line
Supra‐arcuate	12/73 (16.4%)	1.5 ± 0.4 cm anterior to the postglenoid process

**FIGURE 2:**
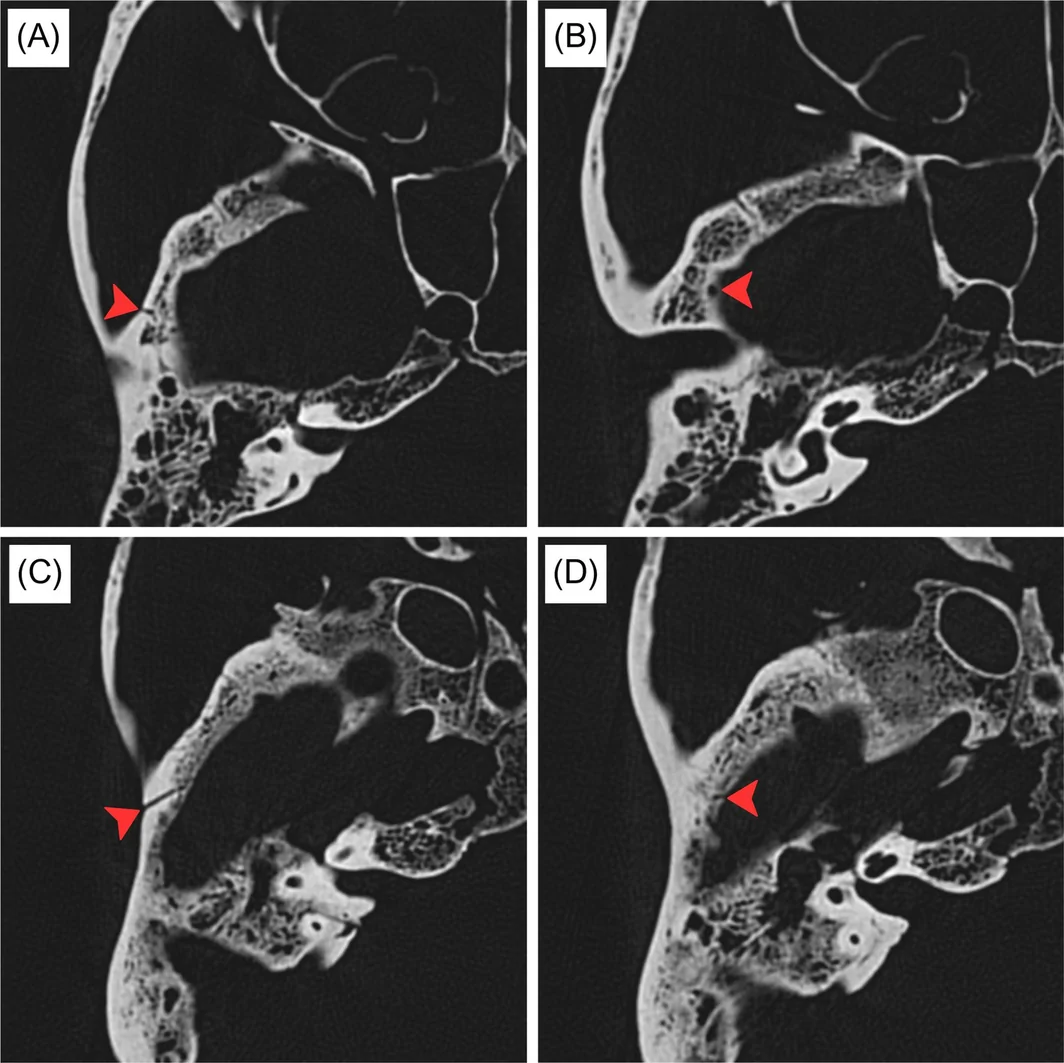
Axial computed tomography images showing the anterior type (A, B) and posterior type (C, D) of the foramen of the squamous part of the temporal bone. The opening from the outer surface of the temporal bone (A, C) and the corresponding opening from the inner surface (B, D) are indicated by the red arrowheads.

In two cadaveric skulls where a foramen was identified, it was completely covered by periosteum and contained no visible structures. The foramen became apparent only after the periosteum was carefully removed (Figure 1D). Communication with the middle cranial fossa was observed in 3 of the 117 skulls; the remaining foramina were non‐communicating (Figure 2). CT imaging confirmed cranial communication in the three cases (Figure 2). In all three cases, there was a connection from the squamous part of the temporal bone to the middle cranial fossa of the cranial cavity.

## 4 Discussion

Although the foremen of the squamous part of the temporal bone has been documented in the literature as PGF, its definition and precise location have remained ambiguous. This study clarifies its variability and proposes a classification based on anatomical position. Notably, this is the first study to examine this foramen in a Thai population. In our sample, the foramen was present in 41.0% of skulls—substantially higher than previously reported rates including 0.6% (Boyd 1930), 3.5% (Wysocki 2002) and 2% (Priya and Yasodai 2020). The unilateral occurrence found in most cases aligns with earlier observations by Conroy. Previous studies did not distinguish foramina based on location and uniformly referred to them as “postglenoid foramen.” However, anatomically, the historical term “postglenoid” should apply only to foramina located posterior to the glenoid fossa. To address this issue, we propose a classification based on position: preglenoid, postglenoid, glenoid, and supra‐arcuate. This system enhances clarity and reflects actual anatomical variation.

During prenatal development, the temporal bone arises from five distinct components: the squamous, styloid, mastoid, tympanic, and petrous parts (Nada et al. 2021). The squamous part, along with the smaller anterosuperior portion of the mastoid segment, undergoes intramembranous ossification. In contrast, the remaining regions, including the larger posteroinferior portion of the mastoid, form part of the cranial base, originate from the otic capsule, and ossify through an endochondral process (Jin et al. 2016; Lupu et al. 2010). Overall, the temporal bone develops from ten primary ossification centers, which are organized around key anatomical landmarks such as the cranial nerves, internal acoustic opening, and the anterior, posterior, and lateral semicircular canals (Nemzek et al. 2000). With regard to the squamous part of the temporal bone, Grzonkowska et al. (2023) found that the primary ossification center of the squamous part of the temporal bone grows linearly in respect to its vertical diameter and according to fourth‐degree polynomial functions in respect to its sagittal diameter, projection surface area, and volume. We hypothesize that when a neurovascular structure, PSS included, persists beyond birth, it may inhibit complete ossification of the surrounding bone, resulting in the formation of a persistent, communicating foramen as observed in the present study.

Although further studies are needed to confirm the contents of these foramina, several hypotheses have been proposed regarding the structures that occupy these foramina. It is generally accepted that the PSS passes through the PGF. However, in the present study, the observed prevalence of the PGF was approximately 30%, which is notably lower than the reported prevalence of the PSS (65%) in a study by Giesemann et al. (2011). This discrepancy suggests that the presence of the PSS does not necessarily imply communication through these foramina. Furthermore, we found that the postglenoid type, anatomically corresponding to the traditional PGF, was present in only 6.0% of cases. It is important to note, however, that their study (Giesemann et al. 2011) was conducted in patients with semicircular canal aplasia (SCA), and their reported prevalence of the PSS may not be generalizable to the broader population. Supporting this, a systematic review and meta‐analysis by Pękala et al. (2018) reported that the estimated prevalence of the PSS in normal adults was only 11.1%. Over half of the patients with SCA were reported to have CHARGE syndrome (Satar et al. 2003). This condition is characterized by multiple craniofacial malformations, including defects in the temporal bone and inner ear structures, which may disrupt the normal pattern of venous drainage. As a result, we hypothesize that primitive embryonic channels such as the PSS may persist to accommodate compensatory venous outflow. Another possible structure that may traverse the foramen is the middle meningeal artery. In some cases, a superiorly projecting groove was observed in association with the foramen (Figure 1B), resembling the typical groove formed by the middle meningeal artery (Sakamoto 2021). This morphological similarity raises the possibility that, in certain individuals, the artery may follow an atypical course. Indeed, previous reports have documented cases where the foramen spinosum was absent (Özdemir et al. 2024), which can be accompanied by an abnormal trajectory of the middle meningeal artery (Nikolova et al. 2012). These findings suggest that the artery does not always pass through the foramen spinosum and may, instead, utilize alternative pathways such as the foramina that communicate through‐and‐through as observed in this study. Also, we cannot neglect the possibility that the foramina observed in our study could simply be nutrient foramina, containing a branch of nutrient vessels arising from the adjacent blood vessels. In addition, we identified three cases in which the PGF appeared to connect the squamous part of the temporal bone to the middle cranial fossa (Figure 2). It is plausible that such a communicating foramen could facilitate the transmission of infections from extracranial to intracranial compartments, thereby posing a clinical risk. One well‐documented pathway involves the venous drainage from the so‐called danger triangle of the face (Pannu et al. 2017), particularly the nose and upper lip, which drains into the cavernous sinus, providing a potential route for extracranial infections to spread directly into the cranial cavity. Another extra‐intracranial connection is the cerebrospinal venous system, a valveless network that permits bidirectional flow between the intracranial and spinal compartments (Tobinick and Vega 2006). Thus, there is a possibility that the foramina of the squamous part of the temporal bone might have a similar function. Finally, in some cadaveric skulls, the foramen was completely covered by periosteum and contained no discernible structures (Figure 1D). This finding supports the possibility that such foramina may represent remnants of prenatal circulation, as previously discussed.

This study has several limitations. First, the sample was limited to skulls available from a single anatomical collection in Thailand, which may not represent the full anatomical diversity of other populations. Second, while CT imaging was used to assess cranial communication in selected cases, not all foramina were evaluated with advanced imaging or histological analysis, which may limit conclusions about their internal contents and function. Further research should explore the developmental and histological characteristics of these foramina to better understand their function and potential clinical implications.

## 5 Conclusion

This study examined the morphological variability of foramina located on the squamous part of the temporal bone and proposed a classification system based on anatomical position. The observed prevalence was considerably higher than previously reported, and the foramina were found in diverse locations. These findings suggest that the traditional term “postglenoid foramen” may not adequately describe the full anatomical spectrum.

## Data Availability

The data that support the findings of this study are available from the corresponding author upon reasonable request.
